# Electroactive Shape Memory Property of a Cu-decorated CNT Dispersed PLA/ESO Nanocomposite

**DOI:** 10.3390/ma8095313

**Published:** 2015-09-18

**Authors:** Javed Alam, Aslam Khan, Manawwer Alam, Raja Mohan

**Affiliations:** 1 King Abdullah Institute for Nanotechnology, King Saud University, P.O. Box 2455, Riyadh 11451, Saudi Arabia; E-Mail: aslamkhan@ksu.edu.sa; 2 Research Center, College of Science, King Saud University, Riyadh 11451, Saudi Arabia; E-Mail: malamiitd@gmail.com; 3 School of Engineering and Technology, Jagran Lakecity University, Mugaliyachap, Bhopal 462044, Madhya Pradesh, India; E-Mail: mohanraja27@gmail.com

**Keywords:** shape memory polymer, polylactic acid, nanocomposites, electro actuation, shape recovery

## Abstract

Shape memory polymer (SMP) nanocomposites with a fast electro-actuation speed were prepared by dispersing Cu-decorated carbon nanotubes (CNTs) (Cu-CNTs, 1 wt %, 2 wt %, and 3 wt %) in a polylactic acid (PLA)/epoxidized soybean oil (ESO) blend matrix. The shape memory effect (SME) induced by an electrical current was investigated by a fold-deploy “U”-shape bending test. In addition, the Cu-CNT dispersed PLA/ESO nanocomposite was characterized by atomic force microscopy (AFM), dynamic mechanical analysis (DMA) and tensile and electrical measurements. The results demonstrated that the SME was dependent on the Cu-CNT content in the nanocomposites. When comparing the SMEs of the nanocomposite specimens with different Cu-CNT contents, the 2 wt % Cu-CNT dispersed system exhibited a shape recovery as high as 98% within 35 s due to its higher electrical conductivity that results from uniform Cu-CNT dispersion. However, the nanocomposites that contained 1 wt % and 3 wt % Cu-CNTs required 75 s and 63 s, respectively, to reach a maximum recovery level. In addition, the specimens exhibited better mechanical properties after the addition of Cu-CNTs.

## 1. Introduction

Shape memory polymers (SMPs) and their nanocomposites belong to a class of smart materials known for their ability to return from a deformed state (temporary shape) to their original (permanent) shape upon exposure to an external stimulus [1,2,3,4,5,6,7,8]. SMPs have been applied in deployable structures, morphing structures, biomaterials and biomedical devices, actuators, smart textiles and fabrics, foams, and self-healing and health monitoring applications [9,10,11,12,13,14,15,16,17,18,19,20]. Based on the types of stimulus applied to induce the shape memory effect (SME), the three main categories are as follows: thermo-active (heating sources include inductive heating, joule heating/electrical heating, and mechanical heating and light heating), photo-active (heating source include; irradiation with different wavelengths) and chemo-responsive (chemicals, such as water, and solvents) [21,22,23]. Among these categories, the SMP nanocomposites whose SME is induced by joule heating using electricity have been at the forefront of SM materials science and technology research over the past decade [24,25,26]. In comparison to the direct heating approaches, this innovative heating method has many advantages, such as convenient, uniform heating and remotely controllable, which is especially useful for applications where direct heating is not possible (e.g., self-deployable aerospace structures, actuators, and implanted biomedical devices). For joule heating, SM polymeric materials must be electrically conductive. Therefore, the polymers were filled with sufficient electrical conductive nanofillers. The conductive nanofillers that have been extensively reported in the literature include carbon nanotubes (CNTs), metallic powders, conducting polymers, and carbon fibers. Among them, CNTs and functionalized CNTs with some organic groups such as carboxylic, amine and hydroxyl groups have come more and more into the focus in recent years [27,28,29,30]. Alternatively, metals including Ag, Cu, and Pt decorated CNTs have also attracted significant interest for their superior electrical and thermal properties, which have dominant role to facilitate the fast actuation in SMP nanocomposite induced by electrically resistive heating. Recently, Mohan *et al.* investigated the polymer nanocomposite made of polyurethane (PU) and Ag and Cu decorated CNTs and reported the electrically resistive heating-driven shape-memory effect of the PU/M-CNTs nanocomposite system. In the investigation, they found that Ag and Cu decorated CNTs reinforced polyurethane (PU) nanocomposite exhibited remarkable recoverability of its shape at lower applied dc voltages due to a significant improvement in the thermal and electrical conductivity of the PU/M-CNTs nanocomposite.

In the current study, we used polylactic acid (PLA) instead of polyurethane (PU) as host matrix to prepare electroactive SMP nanocomposite. In comparison to PU, PLA has the advantages of being renewable, biodegradable, biocompatible and inexpensive; as such it has attracted a great industrial and academic attention in recent years. Briefly for the preparation Cu-CNT dispersed PLA/ESO nanocomposite, the so called electroactive SMP nanocomposite, PLA was first blended with epoxidized soybean oil (ESO) to overcome an inherent brittleness of PLA. Then, Cu decorated CNTs (Cu-CNTs) were incorporated into the PLA/ESO blend at different loading ratios to form electrically sensitive structure suitable for electrical resistive heating. Special focus in the study was given on the Cu-CNT contents (1 wt %, 2 wt %, and 3 wt %) that affect the electrical conductivity of the nanocomposites, which is a most dominant factor determining the electro activation. In addition, the influence of the Cu-CNT addition on mechanical, thermal, and morphological properties of the nanocomposite was addressed. The results of the prepared electroactive SMP nanocomposite are reported and discussed in the following sections.

## 2. Experimental

### 2.1. Materials and Instrumentations

#### 2.1.1. Decoration of CNTs with Cu

One gram of carboxylic group functionalized carbon nanotubes (COOH-CNTs; purity: >99 wt %) that were purchased from Grafen Chemical Industries (Ankara, Turkey) were dispersed in 250 mL of MQ water. Then, 250 mL of a 0.25 M copper chloride (CuCl_2_) solution was added to this dispersant and heated for 3 h at 80 ± 5 °C with constant stirring. Next, the (CNTs-COO)^2^^⊖^Cu^2^^⊕^ solid products were collected by centrifugation and dried under vacuum at 60 °C. During the drying process, copper ions developed on the COOH-CNTs and adhered on the surface due to van der Waals forces, as previously reported [31,32].

#### 2.1.2. Preparation of Cu-CNT Dispersed PLA/ESO Nanocomposite

Commercial grade PLA-05 with a density of 1.25 g/cm^3^ that was purchased from Grafen Chemical Industries (Ankara, Turkey) was mixed with epoxidized soybean oil purchased from Sigma Aldrich (St. Louis, MO, USA) in a PLA/ESO weight ratio of 90/10 via solution blending using chloroform as a mutual solvent. After completion of the blending, the Cu-CNTs were added to the PLA/ESO matrix at three different weight percentages (*i.e.*, 1%, 2%, and 3%) and stirred for 3 h. A uniform dispersion of Cu-CNTs in the nanocomposite matrix was obtained by repeating the ultrasonication process at ambient temperature. The prepared nanocomposite solution was transferred to a conical flask and maintained at room temperature to remove air bubbles from the casting solution. Then, the solution was poured into a glass substrate frame with a 7 mm boundary wall. After the evaporation of chloroform, a flexible, non-fractured nanocomposite sheet was formed, which was maintained in a vacuum oven for 1 h at 35 °C to permit the complete evaporation of the chloroform within the samples.

#### 2.1.3. Atomic Force Microscopy

Atomic force microscopy (NX10, AFM Park systems, Suwon, Korea) was used to study the nanofiller-polymer matrix interactions as well as the surface topography of the samples. Images were recorded in the noncontact mode under ambient conditions at a scan frequency of 0.999 Hz.

#### 2.1.4. Mechanical Analysis

The mechanical properties of the dumbbell-shaped specimens were studied using a Lloyd-LR5KPlus (Lloyd Materials Testing, West Sussex, UK) with a load cell capacity of 5 kN. The test was performed at a crosshead speed of 10 mm/min. The tensile data were analysed using the NEXYGEN Plus software.

#### 2.1.5. Dynamic Mechanical Analysis

Dynamic mechanical analysis (DMA/SDTA861e; Mettler Toledo, Zurich, Switzerland) was used to investigate the thermomechanical properties including the storage modulus (E’) and loss modulus (E”) as a function of temperature. All of the measurements were performed at a frequency of 1 Hz and a strain rate of 0.1% using the dual cantilever method with a heating rate of 3 °C/min, and the temperature ranged from −20–120 °C. In addition, the electrical conductivity of the nanocomposites was measured using the four probe method.

#### 2.1.6. Electro active Shape Memory Study

Finally, the electroactive-shape memory experiment was conducted using a bending test, and the recovery process was recorded with a common camera. The samples were prepared as follows: (1) The samples were bent into a “U” shape at a temperature of T_high_ > T_g_ (*i.e.*, 85 ± 1 °C). Then, the temporary shape was quenched at a temperature of T_low_ < T_g_ (*i.e.*, 5 ± 1 °C) using cold water, and the angle of the fixed shape was recorded at 60 ± 1° when the external stress was removed. The recovery rate was obtained from the recovery of the permanent shape after the specimen was reheated by Joule heating in the circuit. Finally, the values of shape recovery ratio (Rr) were calculated using the following equations:

(1) $$\text{Rr \%}=\frac{\text{Shape fixity angle }\left(\theta {}^{\circ}\right)-\text{Shape recovery angle }\left(\theta {}^{\circ}\right)}{\text{shape fixity angle }\left(\theta {}^{\circ}\right)}\times 100\;$$

## 3. Results and Discussion

**Figure 1:**
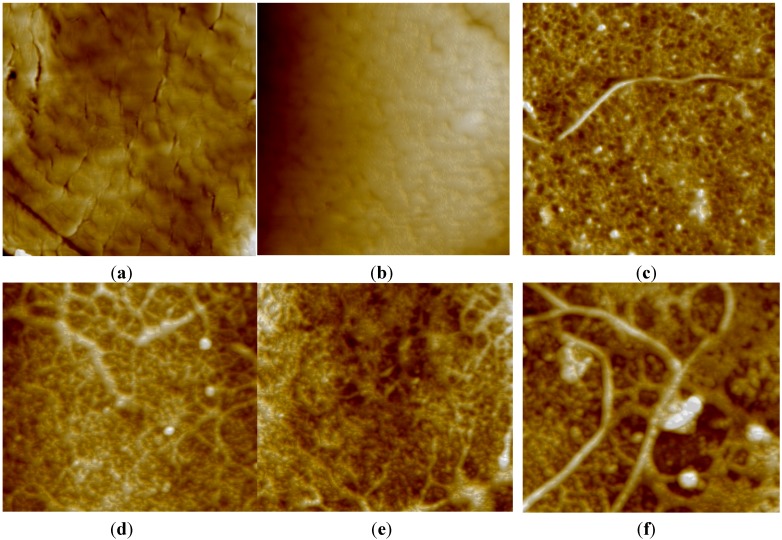
(**a**) Atomic force microscopy (AFM) images of the neat polylactic acid (PLA); (**b**) PLA/epoxidized soybean oil (ESO) blend; (**c**) 1 wt % Cu-carbon nanotube (CNT) dispersed PLA/ESO nanocomposite; (**d**) 2 wt % Cu-CNT dispersed PLA/ESO nanocomposite; (**e**) 3 wt % Cu-CNT dispersed PLA/ESO nanocomposite; (**f**) and 3 wt % Cu-CNT dispersed PLA/ESO nanocomposite.

The Cu decorated CNT-polymer matrix interactions were investigated using AFM, and the results are shown in Figure 1. In the AFM images, the neat PLA exhibited a fractured surface due to its brittle nature. The brittle cracks disappeared when the ESO plasticizer was blended with PLA due to a good plasticizing efficiency of ESO. The incorporation of Cu-CNTs altered the surface consistency of the specimen, which was different from that of the pure PLA and PLA/ESO blend. As shown in the AFM images, the nanocomposite specimen with a filler content of 1 wt % and 2 wt % exhibited a continuous and dense surface where the CNTs were uniformly dispersed. However, as the concentration of CNTs increased from 2–3 wt %, the formation of rigidified polymer layers at the filler-matrix interface was observed due to Cu-CNT agglomeration. In addition, at high filler loadings, the surface texture of the nanocomposite consisted of a two-phase structure consisting of bright nodular domains and dark interstitial regions, indicating that the bulk microstructure of the nanocomposite samples was heterogeneous. The compact and dense surface of the nanocomposite with 2 wt % Cu-CNTs was due to the uniform dispersion of CNTs, which resulted in a greater matrix-filler interfacial area and an enhanced matrix-CNT interface contact.

**Figure 2:**
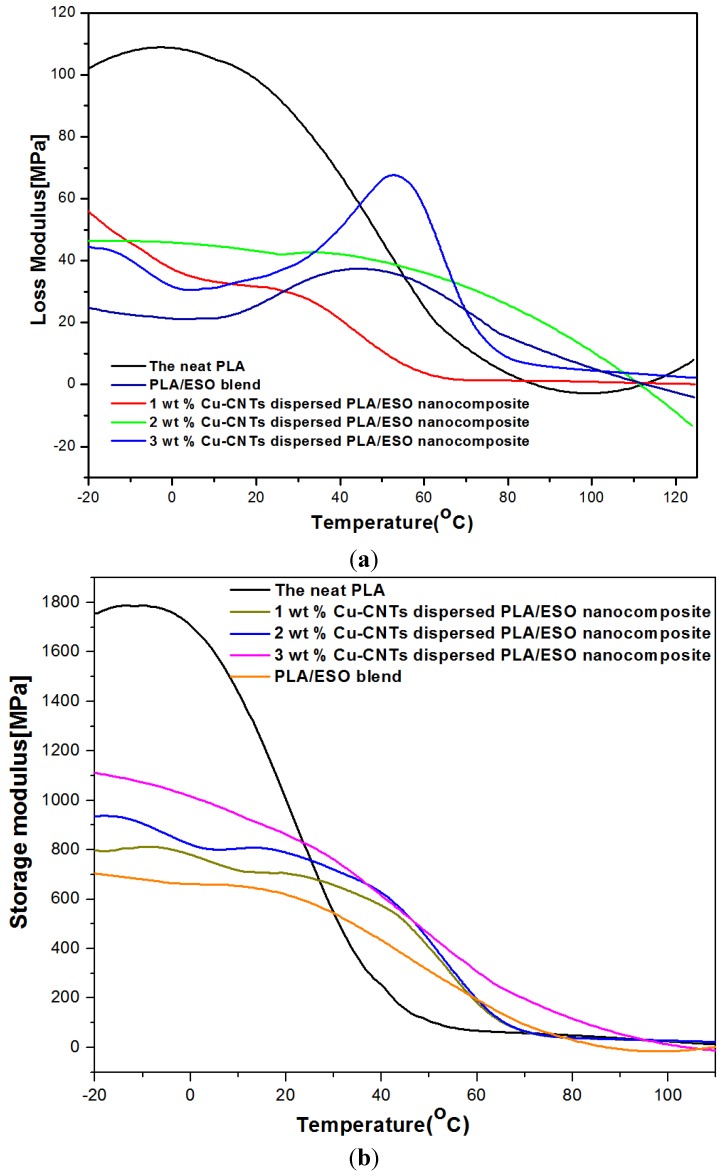
(**a**) Dynamic mechanical analysis (DMA) loss modulus of the as-prepared samples; (**b**) DMA storage modulus of the as-prepared samples.

**Figure 3:**
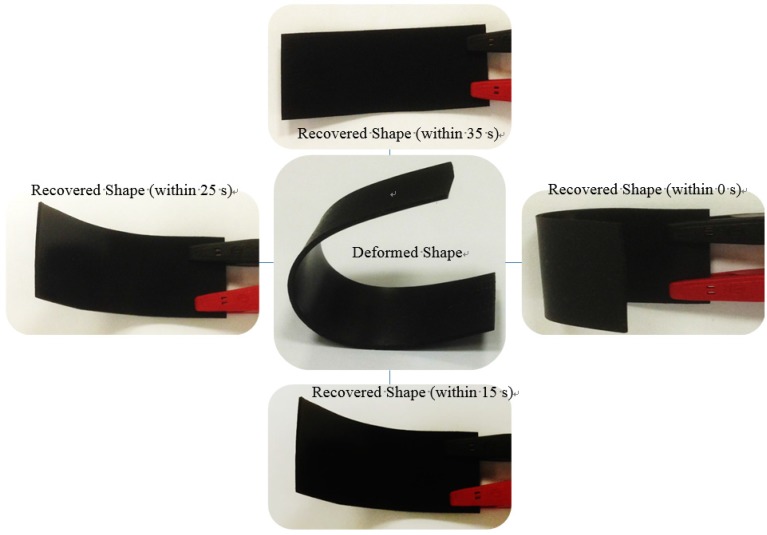
Electroactive shape recovery behaviour of the Cu-CNT dispersed PLA/ESO nanocomposite (Cu-CNTs content of 2 wt %) at a DC voltage of 40 V.

Figure 2 and Figure 3 show the DMA storage modulus (E’) and loss modulus (E”) of the neat PLA, PLA/ESO blend and Cu-CNT filled PLA/ESO nanocomposites as a function of temperature. As expected, the addition of ESO to PLA reduced both E’ and E” due to intermolecular and intramolecular interactions that altered the physical state of the PLA.

However, the addition of Cu-CNTs resulted in an increase in both E’ and E” due to the loss of mobility in the chain segments of the matrix chains resulting from an increased Cu decorated CNT-matrix interaction. The storage modulus increased upon addition of 1–2 wt % CNTs and decreased substantially when an increased CNT content of 3 wt % was added. This significant decrease was due to CNT agglomeration. In general, as the nanofiller concentration increases, the number of nanofillers in the nanocomposite increases, which causes an increased particle-particle interaction rather than a filler-matrix interaction. Therefore, the fillers start to form agglomerates, which ultimately affect the van der Waals interactions between the polymer chains that may result in a decrease in the DMA properties. The large decrease in the DMA properties of the nanocomposite at a higher filler loading was due to a higher level of agglomerated particles that act as defect sites.

In addition, the addition of ESO and Cu-CNTs changed the mechanical properties of PLA, and the results are given in the Table 1. The results indicate that neat PLA was very brittle based on its low elongation value. However, a significant increase in elongation occurred upon addition of ESO to the PLA matrix, confirming the plasticizing effects of ESO. The addition of CNTs to the PLA/ESO blend increased the tensile break and decreased the flexibility of the resulting nanocomposites.

**Table 1:** Mechanical, electrical, and electroactive shape memory properties of the as-prepared samples.

Samples	Stress at break (MPa)	Elongation at break %	Electrical resistivity (Ohm.cm)	Shape recovery time (s)	Shape recovery (%)
Neat PLA	0.63 ± 0.1	7	1.67 × 10^9^	No response	No response
PLA/ESO blend	0.47 ± 0.1	135	7.5 × 10^9^	No response	No response
1 wt % Cu-CNT dispersed nanocomposite	0.90 ± 0.1	45	1.1 × 10^4^	75	47
2 wt % Cu-CNT dispersed nanocomposite	0.97 ± 0.1	39.5	1.9 × 10^2^	35	98
3 wt % Cu-CNT dispersed nanocomposite	0.85 ± 0.1	11.3	3.5 × 10^3^	63	53

However, an increase in the CNT content above 2 wt % led to a significant decrease in the mechanical properties due to the increased agglomeration of the CNTs. The Cu-CNT dispersed PLA/ESO nanocomposite at Cu-CNT loading of 2 wt % exhibited optimal mechanical properties due to uniformly dispersed CNTs, which improved CNTs-PLA/ESO matrix adhesion. This adhesion typically plays a significant role in transferring stress and elastic deformation from the matrix to the fillers.

Finally, the electroactive SM performance of the nanocomposites containing three different wt % (1 wt %, 2 wt %, and 3 wt %) of the Cu-CNTs at an applied constant DC voltage (40 V) was investigated. The rectangular nanocomposite specimens were deformed into a “U” shaped structure by heating the samples in hot water at 85 °C for 45 s followed by rapid quenching in cold water. Then, the shape recovery was recorded using a camera, and the results are shown in the table. The results indicated that the 2 wt % filled SMP nanocomposite exhibited a prompt shape recovery response due to more efficient and fast heat conduction through the nanocomposite, and this nanocomposite recovered its original shape within 35 s (Figure 3). However, the nanocomposites containing 1 and 3 wt % Cu-CNTs exhibited the maximum shape recovery in 75 s and 63 s, receptively. The relatively fast rate of actuation in the nanocomposite with 2 wt % Cu-CNTs may be due to higher electrical conductivity resulting from a uniform Cu-CNT dispersion in the nanocomposite. Uniformly dispersed nanofillers in matrices typically decrease the interfacial electrical contact resistance between the fillers and the surrounding polymer matrix, which leads to the transfer of electrons dominating the joule heating within the polymer nanocomposite. However, particle agglomeration can cause an increase in particle-particle interactions rather than particle-matrix interactions, which deteriorates the carrier mobility and leads to a decrease in the electric conductivity (as can be seen in the table). In addition, the nanocomposite with less than 2 wt % Cu-CNTs exhibited a slow recovery. Therefore, a Cu-CNT loading of less than 2 wt % does not result in the formation of sufficient continuous electrically conductive networks in the nanocomposite.

## 4. Conclusions

Advanced SMP nanocomposites containing different weight percentages (1 wt %, 2 wt %, and 3 wt %) of Cu-CNTs and the PLA/ESO blend were prepared by solution dispersion blending followed by a solvent casting technique. Based on the results, the incorporation of Cu-CNTs led to a significant improvement in the electrical characteristics of the nanocomposite at a very low filler loading, which contributed to a fast shape recovery response. The nanocomposite containing 3 wt % Cu-CNTs exhibited a shape recovery of 98% within 35 s. However, as the filler loading increased from 2 wt % to 3 wt %, the shape recovery decreased due to particle agglomeration. In addition, the incorporation of less than 2 wt % Cu-CNTs did not result in the formation of sufficient continuous electrical conductive networks in the nanocomposite, which resulted in a marginal response to the shape recovery. Moreover, the nanocomposite specimens exhibited better mechanical properties after the addition of Cu-CNTs.
